# Reforming medical device regulation in Europe: centralization, governance, and the case for an EU medical device agency

**DOI:** 10.1016/j.lanepe.2026.101908

**Published:** 2026-10-28

**Authors:** Anett Schönfelder, Tom Melvin, Timo Minssen, Stephen Gilbert

**Affiliations:** a Else Kroener Fresenius Center for Digital Health, Faculty of Medicine and University Hospital Carl Gustav Carus, TUD Dresden University of Technology, Dresden, Germany; b Institute for Clinical Trials, College of Medicine Nursing and Health Sciences, University of Galway, Galway, Ireland; c Center for Advanced Studies in Bioscience Innovation Law (CeBIL), Faculty of Law, University of Copenhagen, Copenhagen, Denmark; d Faculty of Business and Economics, TUD Dresden University of Technology, Dresden, Germany

**Keywords:** Health policy, European MedTech, Medical device regulation, Medical device EU agency

## Abstract

Innovation in medical technologies, especially AI-enabled tools, is exposing the limits of Europe’s current medical device regulatory dogma. Existing rules treat devices as isolated products rather than integrated systems shaping care. Any region that wants to be home to the most innovative businesses and technologies must have a regulatory system fit for adopting state-of-the-art technologies and making fast, effective decisions. While US and UK frameworks innovate, Europe’s model stifles medical-technology innovation and the exploration of new regulatory paradigms through ineffective multi-level governance and uncoordinated, decentralized decision-making. In response, MedTech stakeholders, including industry, professional associations, patient representatives, hospitals, politicians, national authorities, and regulatory scientists, have recently called for a centralised EU-level “single-authority” (for strategy-setting, coordination, and regulatory support) to take accountable ownership of the regulatory system and its strategic development for all medical technologies in Europe. We describe the benefits and drawbacks of this approach and contend how centralization in the complex MedTech system could actually work.

## A complex web: how the current European MedTech system operates

Medical Technologies are essential for delivering healthcare, as they enable the diagnosis, prevention, monitoring, prediction, treatment or alleviation of diseases, disabilities or injuries.^1^ While their use as a hardware device, in-vitro diagnostic device or digital tools/software is accompanied by inherent risks that can result in patient harm, they are covered by comprehensive regulatory frameworks. In Europe, MedTech products are primarily governed by the Medical Device Regulation (MDR) 2017/745^1^ and the In Vitro Diagnostic Medical Device Regulation (IVDR) 2017/746,^2^ which are based on a risk class system according to their potential impact on patients and users. Prior to market approval, manufacturers must demonstrate that the MDR/IVDR requirements have been met based on conformity assessment. For certain devices, this procedure involves designated third-party entities (Notified Bodies (NB)). On completion, manufacturers can apply the CE marking, which is currently valid in over 100 jurisdictions around the world.^3^

Beyond manufacturers and NB, several additional actors influence the process, either directly or indirectly. They operate within a complicated, multi-organisational framework (as shown in Fig. 1), where authority and information flow across EU-level bodies, national authorities, designated private actors, and interest groups, spanning: (i) the EU level, including the European Commission (EC), which chairs the Medical Device Coordination Group (MDCG) and initiates and coordinates relevant projects in the field, managed via DG SANTE. The EC also can initiate new legislation under the ‘ordinary legislative procedure’ and prepare legislative proposals, involving the European Council and Parliament in the following legislative process. The MDCG, composed of Member State representatives and chaired by the EC, is responsible for guidance, standard development, and harmonization of the framework’s implementation, as well as dealing with key sectoral issues, such as oversight of NB. The European Medicines Agency (EMA) is primarily mandated to co-ordinate the regulation of pharmaceuticals. For drug-device combination products the EMA may provide opinions on the medicinal product aspects. The EMA also provides technical and scientific support to medical device expert panels (EXPAMED), which deliver scientific opinions to NB during the conformity assessment of certain high-risk devices under MDR/IVDR.^5^ EMA cooperates closely with the national medicines agencies and their heads (HMA), which hosts the HMA Core Group for Medical Devices (CGMD), bringing together HMA and Competent Authorities for Medical Devices (CAMD) representatives; (ii) at the national level, there are 27 National Competent Authorities (NCA) and Designating Authorities (DA) (with delegated regional authorities in some Member States such as Germany) that are responsible for overseeing products on the market (even though they are not directly involved in the pre-market approval decisions) and mandated to inspect NB; (iii) industry includes currently about 53 NB across Europe^6^ that administer the conformity assessments for medical devices and IVDs before they are placed on the market, as well as over 37,000 MedTech companies^7^; and (iv) supporting groups that provide expert knowledge, such as the Joint Research Centre (JRC) and EXPAMED for high-risk devices, or represent stakeholders of the system, for example the Notified Body Coordination Group (NBCG-Med) or CAMD.

**Fig. 1:**
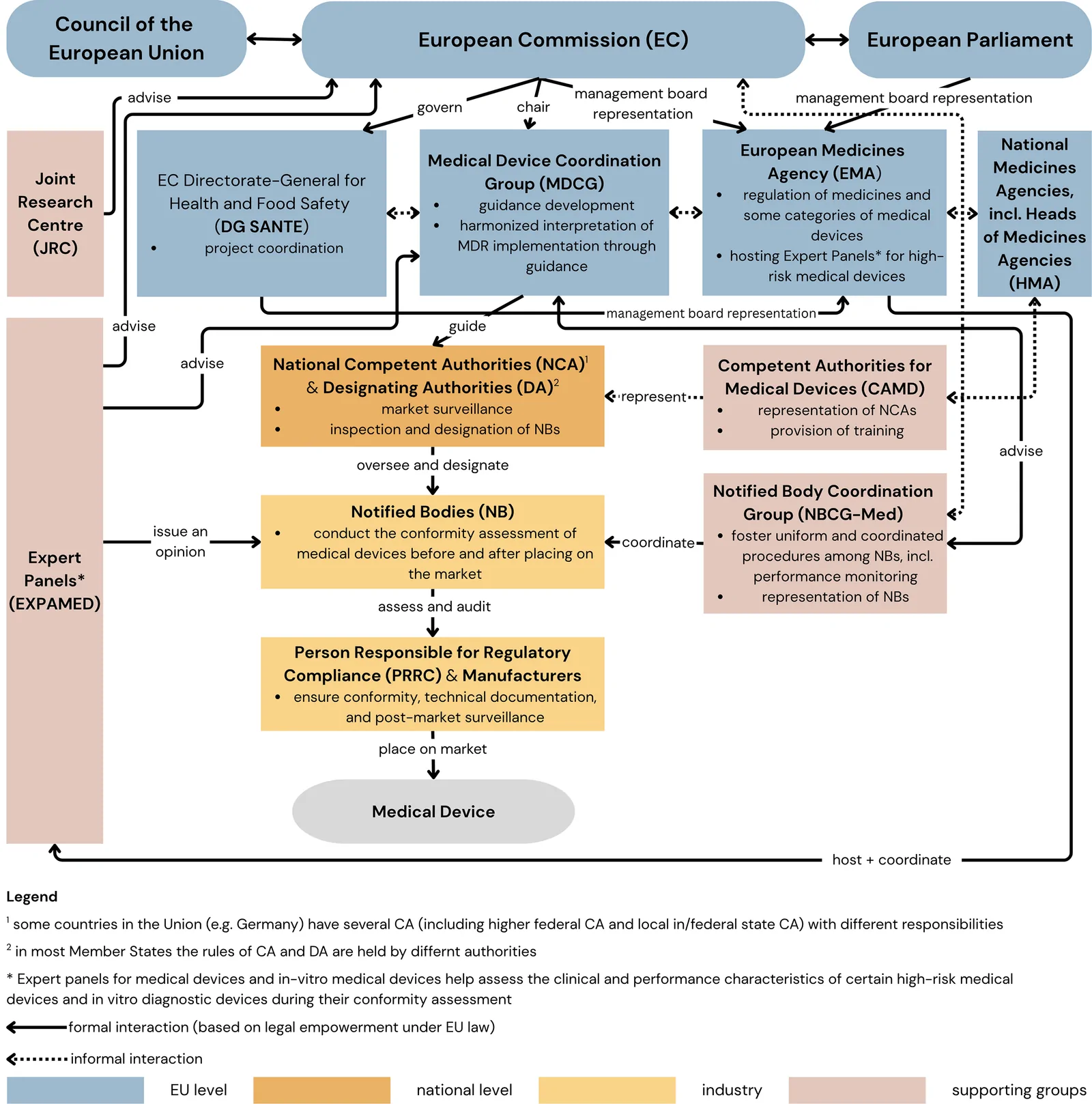
The current medical device regulation system is characterised by multiple layers of governance, where both authority and information are shared and exchanged among multiple stakeholders. However, it is high on bureaucracy, but low transparency and effective communication.^4^

Accompanied by a recent broader shift among European legislators to reduce regulatory burdens and harmonise digital regulations (the Digital Omnibus package^8^ and the Digital Fitness Check^9^), the European Commission published a proposal for simplifying MDR and IVDR on December 16, 2025.^10^ The proposal, which will probably come into force in 2027 once it has passed through the legislative consultation and refinement process, aims to reduce the regulatory burden, including by strengthening the centralisation of conformity assessment processes, and addressing some of the issues outlined below, particularly those concerning NB (e.g., reducing NB involvement for some device types, reducing unannounced audits and the frequency of audits in general, promoting presubmission dialogue and increasing NB regulation and oversight).

## Safety, evidence, and the risks of regulatory divergence

According to the theory of system competition, companies choose their preferred market much like consumers picking between competing products,^11^ pursuing market access in regulatory frameworks considered optimal.^12^ An optimal condition would be to have well-functioning, self-balancing regulatory systems that safeguards patient safety, promotes responsible, technologically-led improvements in healthcare delivery, and enables and sustains a homegrown technology and industrial sector. Such an ecosystem strengthens health systems and, via economic growth and employment, helps generate the revenues that ultimately finance care. In international comparison, it is evident that Europe is losing ground here. For instance, according to a representative survey among 211 medical device and IVD manufacturers conducted in 2024,^3^ they deprioritize the EU as their first regulatory market. The results show that the number of new innovative product launches in the EU has dropped by 33% for large medical device manufacturers and by 19% for small ones, as well as by 40% for large and by 12% for small IVD manufacturers. Meanwhile, the US market is gaining in popularity, particularly among large manufacturers. This is accompanied by a significant decline in innovation activities, especially concerning the development of new devices (59% of IVD and 54,4% of medical device manufacturers) within SMEs.^3^

While there are many factors influencing this development, most of them can be reasoned as systematic shortcomings. In particular, NB and the NB-based system face substantial criticism, regarding their ability to scale capacity at times of change,^13^ how their role is defined,^14^, ^15^, ^16^ and their variability in fees and lack of transparency and speed of procedures.^3^, ^17^ Although the European Commission has enabled transparency regarding the length and cost of the conformity assessments by publishing a list containing links to individual NB fees^18^ and conducting an NB survey including time-related evaluations,^19^ the conformity assessments remain unpredictable, with divergent opinions on similar products and a lack of clarity regarding the evidence expected. For example, empirical study data showed inconsistency in MDR interpretations across NB with non-uniform standards about data required for clinical evaluations^20^, ^21^ and on the handling of conditional certifications for high-risk devices.^22^ Furthermore, data from the CORE-MD (Coordinating Research and Evidence for high-risk Medical Devices) consortium show a significant lack of clinical evidence and transparency: 9% of cardiovascular devices and 0% of orthopaedic implants analysed had any pre-market clinical evidence publicly available at the time of marketing.^23^ This makes it near impossible for clinicians to perform independent risk-benefit assessments before a device is used in a patient, putting patient safety at risk. The introduction of EXPAMED and the Clinical Evaluation Consultation Procedure (CECP) was a political compromise to avoid an agency model of regulation. The opinions published display a marked disparity in the evidence deemed acceptable between regulators and expert clinicians.^24^ EXPAMED have identified critical methodological flaws in the clinical evaluations that NB had accepted, ranging from poor choice of study design, to insufficient sample sizes and poor post-market evidence generation plans.^25^ In all cases, regardless of the flaws identified, the products were granted market approval.

It can be hard for innovators to determine what the EUs stance or approval pathway for a particular technology really is. This becomes especially problematic for new medical devices increasingly taking the form of combination technologies. For example, an AI-powered closed-loop insulin delivery system (“artificial pancreas”), falls under multiple product-related regulatory frameworks, such as medicines (insulin), medical devices (the pump and the continuous glucose monitor), and artificial intelligence (the AI predictive dosing algorithm), such systems could also be linked to patient-used IVD-analysing samples of body fluids (e.g., urine). In practice, manufacturers have to comply with each of these regimes when the device falls within scope. Because parts of these frameworks may appear to overlap in practice (e.g., inconsistencies in qualification and classification,^26^ gaps in interpretation regarding ethical compliance,^27^ and uncertainties concerning assessment roles and responsibilities^28^), this can create unclear and often additional requirements for manufacturers,^29^ as well as slow timelines and conflicting decisions.^30^ Moreover, regulatory uncertainty is directly connected to increased approval times for innovative and novel medical devices, resulting in additional costs.^31^ Acknowledging this, the MDCG and the Joint Artificial Intelligence Board (AIB) explicitly provides a structured approach to avoiding duplication and minimising additional burdens.^32^ Additionally, the Commission’s MDR/IVDR 2.0 proposal together with the proposal for an “European Biotech Act”^33^ aims to provide a clearer categorization of combined products. However, due to divergent national implementation of the regulatory frameworks, it is particularly important for MedTech manufacturers to have access to dialogue formats during pre-submission stages or to general *ad hoc* expertise. Unfortunately, scientific or clinical development advice is not/very limited available from decision makers (NB are explicitly restricted to consultancy activities,^1^ and NCA show divergent practices and knowledge constraints^34^ (e.g., 57% offer advice prior to application, but only 11% offer national scientific advice beyond clinical-trial aspects^35^), and a general lack of coordinated early regulatory advice^34^). In reality, advice is only available from EXPAMED, who have no decision making role in the system. There are no signs of a broader shift in this regard, as recently launched pilot programmes follow the same path, such as EMA’s pilot for “breakthrough” devices^36^: it offers manufacturers priority scientific advice from EXPAMED, but it does not touch the underlying authorisation architecture, therefore adding layers to the unchanged decentralised system with no control on final decisions made by NB. Other jurisdictions, such as the US, are adopting a more courageous approach by linking centralized binding advice and authorization (FDA’s Breakthrough Devices Program) with centralized reimbursement (the newly proposed RAPID Coverage Pathway, which would issue a proposed national coverage determination the same day a breakthrough device receives FDA authorisation)^37^ - something that is currently unthinkable within the multiple layers of overlapping EU decentralization.

Compounding this, the siloed decision-making on a NB-by-NB, device-by-device and manufacturer-by-manufacturer basis yields minimal information sharing or learning mechanisms (e.g., there is no publicly accessible database containing previous NB-decisions^38^), and persists due a leadership vacuum, in which none of the multiple stakeholders (shown in Fig. 1) are mandated to explain their decision-making on scientific, strategic and regulatory themes and for disseminating them throughout the system. In a study conducted by the Directorate-General for Research and Innovation on behalf of the European Commission, all of the MedTech companies interviewed described this as an “accountability vacuum” across fragmented governance levels.^16^ Critically, when various regulatory actors—NB, EXPAMED, and NCA—operate in silos, safety falls into a “no man’s land” where responsibility is diffused, and patient safety is put at risk. However, the systematic shortcomings we highlight relate to the structure of the system that has evolved over time as a result of legislative requirements, and are not a criticism of the motivations, intent, or capabilities of the individuals operating within the system.

## At a breaking point? The call for change in EU medical device regulation

In reaction to the systematically rooted problems, a broad array of stakeholders, including policymakers, national authorities and industry representatives, have recently called for governance reform with the focus of creating a centralised structure at EU-level that is both accountable and capable of taking full ownership of the medical technology regulatory system and its strategy. Position papers have been published by (i) a consortium of industry voices spanning the European trade association for the medical technology industry (MedTech Europe), the Association of the European Self-Care Industry (AESGP), the European Coordination Committee of the Radiological, Electromedical and Healthcare Information Technology Industry (COCIR), the Federation of the European Dental Industry (FIDE), the Federation of High-Tech Industries (EUROM), and the European Association of Authorised Representatives (EEAR)^39^; (ii) an initiative of 35 European medical specialist societies encompassing health professionals and researchers, represented by the Biomedical Alliance in Europe (BioMed Alliance),^38^ both in March 2025; as well as by (iii) 21 European medical device competent authorities represented through the CAMD and HMA networks^40^; and (iv) the NB Coordination Group-Med Executive Committee together with the Team-NB Executive Committee,^41^ both in July 2025; and (iii) representatives of patients and hospitals, represented by the European Patients Forum (EPF), the European Association of Hospital Pharmacists (EAHP), the European Hospital and Healthcare Federation (HOPE) and the Biomedical Alliance in Europe (BioMed Alliance)^42^ in September 2025. Additionally, members of the European Parliament issued a motion for a resolution emphasizing the urgent need to revise the MDR (procedure 2024/2849(RSP)) in October 2024, in which they called for the establishment of a central governance structure.^43^

While each position paper varies regarding the design and implementation of a governance reform and their demand for more or less centralization (Table 1), the common idea is simple: The purpose of introducing a central body would be to consolidate responsibilities and eliminate the current fragmentation across multiple actors. This is reasoned in a missing “captain of the ship”,^39^ a lack of accountability, transparency and stakeholder involvement,^38^, ^39^ and insufficient coordination,^42^ leading to inconsistent rule application and efficiency gaps.^40^, ^41^ While the position papers collectively address all of the above problems, they do so selectively, foregrounding issues that align with each group’s interests and needs, or that are necessary to maintain their strategic autonomy and *status quo*. For example, stakeholders closest to clinical use, such as healthcare professionals, researchers, and patients, centre their arguments on gaps in clinical evidence and transparency, because these deficiencies translate directly into patient-safety and practice-level consequences.^38^, ^42^ By contrast, industry groups emphasize the lack of a single, clearly mandated contact and representative body at EU and international level as this creates fragmented guidance and weakens consistent presentation across Member States and internationally.^38^, ^39^, ^41^

**Table 1:** Comparison of the position paper published with a focus of creating a central EU governance structure for medical devices.

Influencer	Industry (manufacturers)	European authorities	Industry (NB)	Clinicians and researchers	Policy-makers	Hospitals and patients
Author (publication date)	**MedTech Europe, AESGP, COCIR, FIDE, EUROM and EEAR (03/25)**	**CAMD and HMA (07/25)**	**NBCG-Med Executive Committee and Team-NB Executive Committee (07/25)**	**BioMed Alliance (03/25)**	**Members of the European Parliament (10/24)**	**EPF, EAHP, HOPE, and BioMed Alliance (09/25)**
Aspect						
Governance Reform	Yes, missing ‘Captain of the Ship’ and accountability gap affects efficiency in CE-marking, delays device availability, and challenges sectors competitiveness	Yes, fragmentation hindering harmonization and effective practical application	Yes, due to fragmentation, inconsistent rule application, and inefficiencies	Yes, due MDR and IVDR unintended consequences (e.g., lack of special regulatory pathways, limited clinical expert engagement, insufficient transparency of clinical evidence)	Yes, due MDR shortcomings	Yes, due to fragmented oversight and insufficient coordination
Central Structure approach	**New single central governance structure at EU level**	**Central agency**	**Medical Device Coordination Office (MDCO)**	**Medical device division within EMA** (then EMDA - European Medicines and Medical Devices Agency)	**Central governance structure or medical device office within** the Commission’s **Directorate-General for Health**	**Central EU Body for Scientific Co-ordination**
Key tasks, roles and responsibilities	- Set strategy and take decisions for the whole sector - Act as international representative - Ensure alignment and coherence within EU legislation - NB designation, oversight and harmonization - Guidance development	- Enhance regulatory support and promote clarity, consistency, and harmonisation in applying requirements - Support NCA cooperation - NB designation and oversight - Provide regulatory advisory services - Implement adaptive pathways	- Central EU-level coordination, support, and guidance - NB designation, oversight and reassessment - Single representative for stakeholders - Enhancing scientific, regulatory and consultation coordination - Technical and administrative secretariat for MDCG	- Manage EXPAMED scientific opinions and coordinate EU input to international standards/harmonisation (IMDRF/MDSAP) - Strategic coordination of specialist regulatory communities - Monitoring expertise in NCA, designate lead agencies and NB specialisations - Single point of contact for stakeholders - Coordinate clinical trials and manage special pathways	- Centralize authority over NB (designation and surveillance) - Harmonize administrative practices - Guidance development (MDR + IVDR) - Ensure alignment and coherence within EU legislation - Coordinate Member States market surveillance and vigilance activities	- Provide binding advice both clinical and scientific - Standardise evidence requirements - Ensure clinical and scientific evidence application across NB
Role of NB	Retain current tasks under new structure	Retain	Retain as ‘extended arm’ of the authorities	Retain, as alternative options are politically infeasible	Retain	Retain
Clinical/Scientific Input	Implicit through the need of accelerating innovation capacity and competitiveness, and resources for specific expertise	Need for a scientific basis and strong on going support (scientific secretariat)	MDCO responsible for maintaining a structured catalogue for scientific and clinical data sources	Through EU networks of specialist regulators with clinical experience, supported by medical and scientific experts	In terms of a post-marked surveillance system/database using RWD and patient feedback	Medical, scientific, and regulatory experts within the EU body
Funding Model	Implicit need for sufficient budget	Sustainable funding and sufficient and capable resources on EU and national level	Sustainable budget through a hybrid funding model (combination of stakeholder fees linked to EUDAMED and EU budget, option for additional fees for manufacturers advice and NB-services)	Implied within EMA structure	–	–
Next Steps/Implemen-tation	Definition of empowerment level, roles and responsibilities, then impact assessment to evaluate the main organizational options	Detailed plan and resource assessment for governance model improvement and exploration of the potential of centralization and operational coordination	Establishment of the MDCO	Establish EMA medical device division	Influencing later discourse in EU Parliament	Create a Central EU Body for Scientific Co-ordination via MDR revision

EMA: European Medicines Agency; EXPAMED: Expert panels for medical devices and in-vitro medical devices; NB: Notified Bodies; NCA: National Competent Authorities; RWD: real-world data.

Throughout, we distinguish centralization of product authorisation (i.e., whether individual devices are approved by a central body rather than by NB) from centralization of regulatory governance (i.e., who sets strategy, defines standards, NB oversight and systemic accountability). As the position papers and Table 1 show, stakeholders advocate on the latter, a central strategic coordination and improved regulatory support (either through binding advice/consultation or guidance development), and argue for maintaining the shared decision making through keeping the authority of product authorization within the remit of NB.^38^, ^39^, ^40^, ^41^, ^42^, ^43^ The latter also applies for the Commission’s proposal for the simplification of MDR and IVDR, which aims to keep the decentralised process with NB^10^ (perhaps because replacing this layer with public actors is considered politically infeasible). However, this is a crucial point because, as long as individual NB decide whether to approve highly innovative technologies in the EU, while other NB can reach different conclusions on highly similar product submissions, with limited transparency about the basis for these decisions and without setting formal precedents for future decisions, core system problems of disconnected decision-making and unpredictable approvals will remain.

Beyond the stakeholders’ case for clearer accountability, more efficient regulatory decisions and more consistent regulation, the technology governance questions raised in this paper increasingly intersects with the EU’s broader push for technological sovereignty and for regulatory simplification (e.g., the Draghi and Letta calls). Recent high-level agendas and the ‘omnibus’ initiatives argue that Europe must reduce strategic dependencies, build capacity in critical technologies, establish implementation-ready, sector-specific governance, and create the conditions for scale and investment within the Single Market. In that framing, medical technologies are not only a regulated sector, rather they form part of the European Union’s ability to secure resilient supply, trusted data infrastructures, and interoperable standards—all prerequisites for autonomy in health innovation, as well as for technological competitiveness of the European Union.

## The (dis-)advantages of centralization

The stakeholders’ position papers assume that all the current systematic shortcomings would be resolved by establishing a central EU governance structure for medical devices. We argue that an assessment based on theoretical analysis and available empirical scientific research is required to understand whether centralization, i.e., the transfer of decision-making competences and legislative rights^44^ to European level, would lead to better regulatory outcomes, particularly by expanding this perspective to encompass diverse sectors and institutional settings.

Centralization is superior, for example, in the elimination of redundant processes or misguided incentives, and by providing economies of scale. As recently experienced during COVID-19 pandemic, uncertainty regarding the procurement and distribution of essential medical goods have led to moral hazard problems (e.g., hoarding of goods), competition between supposedly equally ranked countries, and corruption,^45^, ^46^ with negative consequences for population health. In the MedTech sector, NCA adopted different approaches regarding the ability of NB to conduct remote audits, resulting in certification delays and restricted patient access to devices in certain Member States.^39^ Against this background, evidence from Denmark and Italy suggests that centralization can be an effective tool for reducing corruption-related inefficiencies and saving costs: by pooling purchasing power and standardizing procurement procedures, centralized systems reduce the discretion individual buyers have over supplier selection, thereby limiting opportunities for both active waste (e.g., corruption, which can inflate costs by up to 11%) and passive waste (e.g., negligence), and enabling negotiation of lower prices through economies of scale.^47^, ^48^, ^49^ This logic is not limited to governmental structures, it is particularly relevant for companies operating in multiple Member States, for whom a single EU-wide procedure can replace parallel national processes. Considering parallels with broader centralization of decision making, this was achieved by the EU trademark system: after the introduction of the EU trademark, the cost for securing EU-wide protection fell markedly compared with separate national filings (about €3160 versus €1,975, later dropped to less than €600).^50^ Another example is EMA’s centralized procedure that enables the authorization of a medicine simultaneously in all 27 Member States with only one submission and one evaluation (particularly for new products there is an assigned lead and two reporting Member States, followed by a decision by the Committee for Medicinal Products for Human Use (CHMP),^51^ while most generic and prescription-free drugs are assessed and authorised at a national level^52^). This helped to reduce duplication in regulation and fixed cost by replacing parallel community authorization and improved transparency,^53^ and saving time as a study demonstrated that joining EMA’s centralized procedure reduced launch delays by 10.9 months.^54^

Centralization also has advantages when cross-border externalities are large, e.g., with public goods, food safety, the environment, or systemic financial risks. In this regard, several sectors experienced better regulatory outcomes either through structural centralization or administrative centralization. For example, again considering parallels from non-medical device areas, in reaction to the Bovine Spongiform Encephalopathy (BSE) crisis, considered to have been caused by fragmented national food safety assessments, a lack of risk regulation and missing science-based decision-making, the European Food Safety Authority (EFSA) was founded in 2002.^55^ Its core role is systematic risk assessment and scientific advice, which has helped to develop regulatory decisions and improve public health (e.g., Salmonella control programs in poultry). According to a study analysing 745 European banks, either supervised by the Single Supervisory Mechanism or by National Supervisory Authorities, showed that banks moved to a centralized supervision system have significantly reduced their credit risk exposures^56^ while increasing profitability.^57^

Centralization has strengths where a single authority or external bargaining powers are needed, as this supports homogeneity in decision-making and improves credibility. Another main advantage is that the centralising authority better accounts for cross-jurisdictional spillovers. However, this comes with a trade-off: the loss of direct political control that decentralization affords citizens and Member States over decision-makers closer to them (so-called “democratic deficit”). This trade-off is formalised in principal–agent/common-agency theory (Tommasi & Weinschelbaum (2007)^58^). Yet the “accountability advantage”^58^ is often invoked as a catch-all argument in favour of decentralization without much nuance. For example, in the article “The Joint-Decision Trap: Lessons from German Federalism and European Integration”, Scharpf (1988)^59^ describes how incentives for human accountability in governance structures are often absent, and how strict policy adherence is prioritised over common-sense flexibility and how agile policy refinement is excluded, even in cases of evidently flawed policy. Meunier & Nicolaïdis’s (1999) article “Who Speaks for Europe?”^60^ supplements this with an analysis of who holds the authority to represent Europe. This is directly applicable to the decentralized EU MedTech sector, where stakeholders complain about a missing “captain”^38^, ^39^, ^40^, ^41^, ^42^, ^43^ and of an accountability vacuum,^16^ arguably because NB operate across jurisdictions while their oversight remains confined to national authorities, detaching democratic control from effects of NB decisions. Furthermore, the inflexible application of procedures by technocrats who are not empowered to change processes, limits regulatory agility. Additionally, the diffusion of responsibility for policy outcomes across multiple players not only reduces political accountability but is also associated with a lower average quality of policymakers.^34^, ^61^ In MedTech, where regulatory paralysis has measurable consequences for patient access to life-improving technologies, the lack of a central authority willing and empowered to take ownership of policy outcomes is not merely a governance inefficiency, it is a structural competitive disadvantage.

However, centralization is not unconditionally efficient. According to Wallace Oates articles on fiscal federalism (1972/1999), decentralization may be superior in situations where preferences are highly heterogeneous (i.e., where stakeholders have substantially different wants from a regulatory regime).^62^, ^63^ Other potential benefits of decentralization include tailoring policy to local preferences supported by the use of local knowledge,^63^, ^64^ however, this does not apply to MedTech CE marking, which allows market access across all of the European Union and the decentralised NB are not necessarily in the same country as either the development or principal use of the medical device. However, centralization would not necessarily resolve existing MedTech specific system shortcomings, such as long approval times and delays to market entry for innovative products, since the current regulatory patchwork of legislation would still apply.^30^

Based on recent developments, the MedTech sector may also face concerns about “over-centralization”, whereby the power of federal subunits is reduced to that of mere “agents” of the central government.^44^ The U.S. offers an instructive reference point: it has long maintained a central federal agency responsible for medical devices, the Food and Drug Administration (FDA). As the FDA sits within the executive branch, the agency’s priorities and approaches inevitably change with administrations, but recent shifts have been exceptional.^65^ Key concerns include rushed reviews and approvals (e.g., relying on a single pivotal clinical trial instead of two^66^), inconsistency with scientific methods and evidence (e.g., expert and advisory panels that appear unbalanced, intransparent, and with financial conflicts^67^), and politics infiltrating decisions on both specific products and product classes.^65^ This is accompanied by layoffs of experts and leadership turnover^65^, ^68^ at a time when expertise in new technologies and approaches is much needed.

Centralization, in itself, is neither a guarantee nor a precondition of a well-functioning regulatory approvals and oversight system for medical devices. What determines outcomes is the existence of robust, enforceable frameworks and standards and effective mechanisms to ensure their consistent implementation. To support an efficient ecosystem for medical device innovation, safety and market availability, decisions on highly innovative technologies must be made by the best available experts collaboratively, with centrally coordinated interactions and consistent processes. These processes must be linked and aligned to produce justifiable, evidence-based, and repeatable decisions, ones that are communicated evenly to all stakeholders and monitored centrally for their effectiveness. Given that no medical device EU legislation since 1990 has been preceded by a scientific evaluation of policy options and their effects on public health,^69^ a fundamental shift is needed: from policy based evidence to evidence based policy.

## How to (or not to) centralize—drafting a possible solution

Considering all arguments, the crucial question is not *whether* to centralize within the complex EU system, it is *how*. We argue that, in order to provide strong binding powers, a specific EU-level-structure would be needed. An EU agency (similar to the EMA, or the EMA itself), i.e., a distinct EU level entity with a clear legal basis would be needed, to take on this role. That said, a move to a fully centralised, “single-authority” EU system would encounter structural legal challenges. Under the “principle of conferral”, the Union can act only within competences conferred by the Treaties (TEU), and the exercise of non-exclusive competences is governed by “subsidiarity and proportionality” (Article 5 TEU).^70^ In public health, Article 168 TEU requires a high level of health protection but also frames Union action as complementary to national policies and reserves key responsibilities for Member States, limiting the scope for blanket centralization absent a carefully chosen legal basis and design.^70^ For example, existing central EU-agencies have limited enforcement powers and are characterized by shared decision-making, such as EMA jointly develops strategies with the Heads of Medicines Agencies (HMA) for the entire EU medicines regulatory network,^71^ and market authorization, and the subsequent pricing and reimbursement are driven by the Member States.^72^ Medical device rules have largely been constructed as internal-market legislation, and replacing Member State roles in designation/supervision of NB with an EU-only regulator would require significant legislative restructuring of the current system in which NB are designated by Member States to perform conformity assessment tasks. In addition, any central EU “FDA-like” body would need to be designed within EU’s administrative-constitutional limits on delegating powers to agencies: under the Meroni line of case law,^73^ agencies may not be granted open-ended policy discretion, but only clearly defined powers subject to effective oversight. Although later jurisprudence has relaxed this constraint, confirming that delegation is permissible where powers are tightly framed by objective criteria and accompanied by robust safeguards (as in the European Securities and Market Authority (ESMA) law case^74^), the design space remains constitutionally bounded.^75^ As a result, this pushes policymakers toward hybrid models (stronger EU coordination, tighter common rules, and more central technical capacity) rather than a simple legal transplant of a fully unitary regulator.

In their position papers, MedTech stakeholders propose several design approaches (as shown in Table 1), ranging from a new single central governance structure or agency (preferred by industry and European authorities), to a division within existing agencies (preferred by clinicians and researchers as well as policymakers), to a coordination office (preferred by NB, hospitals and patient representatives). These different forms of centralization would have different visibility, power, and economical aspects (Table 2). The lack of consensus between stakeholders could be due, at least in part, to the ongoing debate about the democratic legitimacy and accountability of European agencies.^75^ It may also be grounded in the fear that large costs are needed to fund a truly independent agency.^38^ This concern was also present in previous debates before the introduction of the MDR and IVDR, when the Health Committee of the European Parliament (ENVI)^76^ and the Association Internationale de la Mutualité (AIM)^77^ proposed a centralised agency. This did not happen due to opposition from industry,^78^ patient representatives^77^ and the European Economic and Social Committee (EESC),^79^ who opposed it, amongst other things, because of concerns regarding increased costs. Of course, a sufficient budget would be needed to reflect the service the central unit would offer and to ensure long-term stability, however, it depends on the model chosen whether costs would exceed the latest rates. For example, the annual budget of EMA is 600.2 million for 2025,^80^ of which 91,5% is covered through industry fees.^80^ The model of tying payment to specific service could be adapted and linked to EUDAMED activities while simultaneously abolishing other current fees, as proposed by NB,^41^ thus aligns cost-bearing with benefit-capture. The recently published EU Commission proposal retains the current system, which is not entirely decentralized (it combines elements of both approaches, such as a centralized medical device regulation alongside decentralized authorization decisions). The argument for retaining the NB-structure, while aiming for a more coordinated and streamlined oversight of NB, slightly increases the degree of centralized governance in the EU system, as well as the expanded role for the EMA (e.g., to support SMEs and coordination activities among NCA) and the broadened scope of EXPAMED to increase the provision of scientific, technical, clinical, and regulatory advice.

**Table 2:** A comparison of approaches to centralization in the MedTech sector, ranging from a fully centralized federal agency model to a light coordination office.

Approach	Fully centralized federal agency (FDA model) impossible in the EU	New single central EU governance structure (e.g., Medical Device Agency)	Division within an existing agency (such as EMA)	Coordination office
Dimensions				
Governance and authority	Independent high decision- making power with legal binding authority and enforcement over medical device regulatory strategy, guidance, product authorization, and regulatory pathways	Independent high decision- making power over medical device regulatory strategy and guidance; enforcement and product authorization remains with the Member States	Decision-making power depends on role and hierarchy within existing agency, but it will probably cover medical device regulatory strategy and guidance; enforcement and product authorization remains with the Member States	Limited to shared decision-making across currently established institutions (complex NB-System (Fig. 1)); advisory and coordination function
Scope and functions	Control funding, policy, and strategy for medical device regulation; binding guidance and rule making authority; enforcing cross- sector alignment; implementing research and innovation projects; uniform international presentation	Control strategy for medical device regulation; enforcing cross- sector alignment; implementing research and innovation projects; uniform international presentation; expert panels; funding and policy remains with the Member States	Control strategy for medical device regulation; enforcing cross- sector alignment; implementing research 0 and innovation projects; expert panels; shared representation scope with other aspects of the agency; funding and policy remains with the Member States; risk of overlapping responsibilities with other divisions of the agency.	Coordination of activities and information exchange; guidance development; risk of blurred scope of duties
Structural design	Requires sufficient number of policy and research experts, as well as administrative support staff	Requires sufficient number of policy and research experts, as well as administrative support staff	Requires policy and research experts, administrative support staff could be shared within existing agency	Requires policy and research experts, as well as administrative support staff
Budget	A combination of allocated budget funds and user fees for medical device review and advice service; a large amount will probably be needed, but this will likely be cheaper overall due to less duplication	A combination of allocated budget funds and user fees for advice service; sufficient amount be needed, but this will likely be cheaper overall due to less duplication	A combination of allocated budget funds and user fees for advice service; amount depending on size within existing agency and extent of shared resources	Allocated budget funds; smaller amount be needed because of size and scope, but duplication remains
Communication and flexibility	Effective inter-agency and stakeholder connection and communication; standardized information flow and data- sharing; managed adaptation to new circumstances (including advanced technologies or crises)	Effective stakeholder connection and communication throughout EU system; standardized information flow and data- sharing; managed adaptation to new circumstances (including advanced technologies or crises)	Actionable communication depend on visibility within existing agency; standardized information flow and data-sharing using established tools; adaptation to new circumstances (including advanced technologies or crises) within broader scope	Effort to limit informal information channels which is difficult by a lack of access to or power over certain stakeholders; coordination of consultation procedures; only advice on adaptation to new circumstances (including advanced technologies or crises)

The approaches are contrasted across five key dimensions, capturing how each model affects the degree of centralization.

Each centralization approach comes with different threats and opportunities. These are described in Fig. 2, through a risks/opportunities matrix with a consideration of the likelihood of changes and positive or negative impacts of the changes. This helps to assess and classify trade-offs between centralization and decentralization pointed out in the literature, for example the “risk-return” model of institutional choice (Arcuri & Dari-Mattiacci (2010))^81^: while a central medical device agency benefits from pooled expertise (MDA-3, high sectoral impact, likelihood moderate), and thus lowers the frequency of erroneous authorisation decisions, at the same time it risks a single point of failure (MDA-8, high systemic impact, very low likelihood). We explore in this framework the most centralised option possible within the EU, i.e., the option of a medical device agency compared to the alternative option of a coordination office as a mixed governance model. Our analysis highlights that, although the coordination office is the most feasible option as it can easily be implemented within the existing EU framework, it is not the most effective solution for the sector due to its limited scope, functions and enforcement power. While the changes advocated for are likely to bring many advantages, centralization in general is hard, and even harder in the EU due its diversity between Member States, limited enforcement, and stakeholders that have divergent interests. However, the establishment of a coordination office would maintain an overall decentralised system. There is a danger that current proposals (including the Commission’s proposal) could combine the worst aspects of centralised and decentralised approaches (it is very hard to be both centralised and decentralised simultaneously) and stick with a system that fails to support efficient, world-class healthcare.

**Fig. 2:**
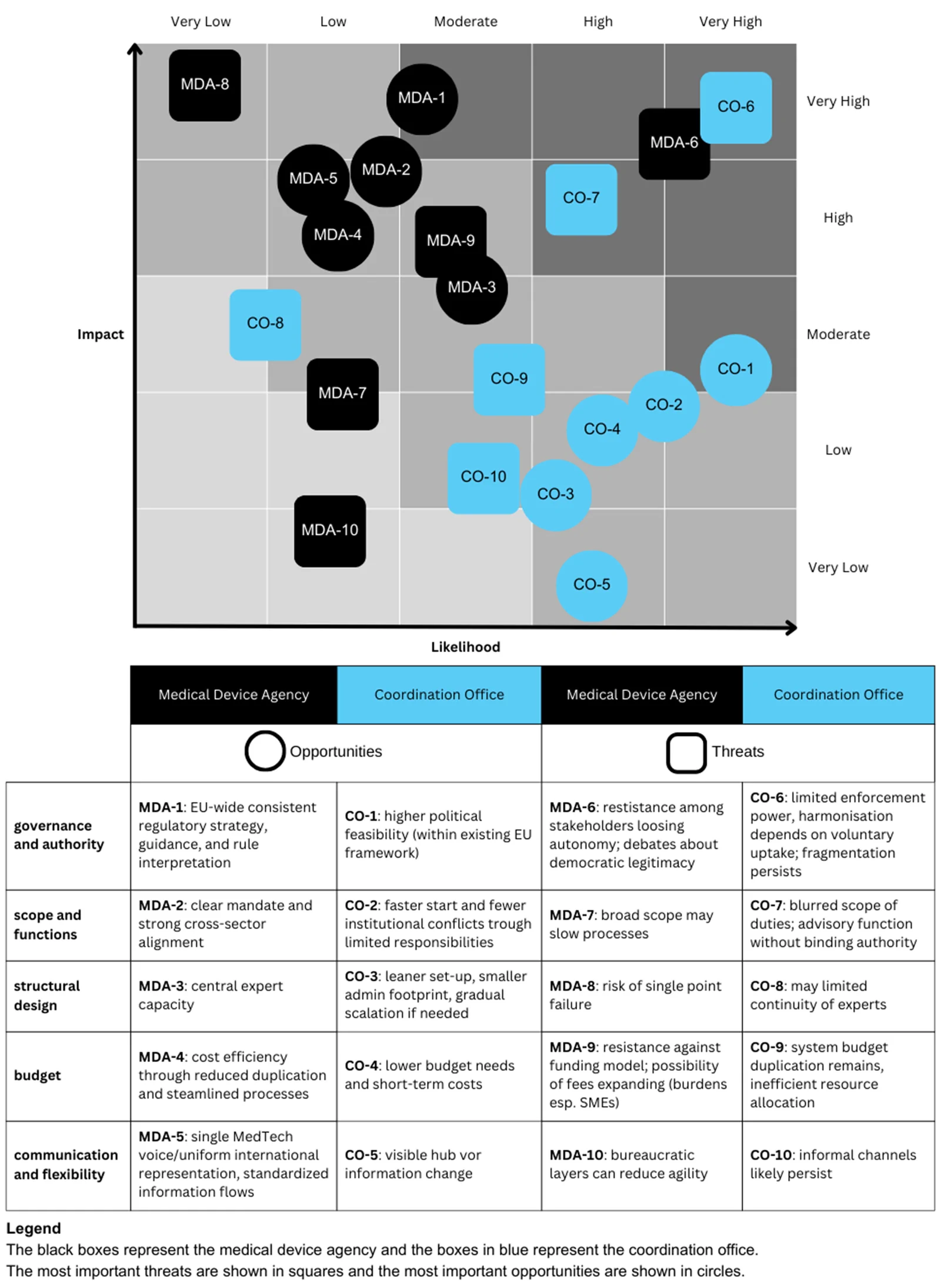
Risks/opportunities matrix comparing threats and opportunities across governance dimensions for the medical device agency and the coordination office approaches, along with the likelihood and impact of changes.

The specific EU-level-structure that we propose is the most centralised option possible within the EU. This could be either a new, single central EU governance structure (e.g., Medical Device Agency), or a well-defined division within an existing agency (such as the EMA), which is equipped with high decision-making power over medical device regulatory strategy and guidance. This unit would need to be empowered both legally and through adequate resources (e.g., adequate subject expert staff with operational experience), and mandated to define and enforce rules, as the US FDA does. Other essential functions that would contribute to a level playing field include: (i) creating general guidance and maintaining a transparent, open-to-all database of existing device classifications; (ii) providing binding, recorded advice on innovative devices and acceptable clinical development strategies, and creating new device classification rules where needed; and (iii) ensuring that decisions on product approvals are evidence-based, and these are propagated as system-level policies which are transparently inherited in all subsequent decisions. To strengthen relationships, cooperation and communication between the central MedTech unit, EU institutions, Member States, industry, and other stakeholders, it would be relevant to establish structured peer-exchange formats and to make information visible, e.g., through dashboard displaying certification throughput, or EUDAMED adoption.

A weakness of previous changes to the EU regulatory governance has been to assume that they will be “right first time”. This is unlikely, and adaptivity of governance structures is needed particularly given the speed and complexity of medical technological development. It is therefore important to allow a fail-fast culture, supported through good, academic-led regulatory science, and to test concepts of networked governance and regulatory work-sharing. For example, the establishment of Centers of Excellence in Regulatory Science and Innovation (CERSIs),^82^ based on the successful US and UK model, could be an option of rather than concentrating all regulatory capacity within a single authority, to establish a reliance and work-sharing model in which academic-led regulatory science provides research of concepts and generates evidence of the effectiveness of regulation^16^ that feeds into and strengthens central decision-making.

## Conclusion

The importance of appropriate and effective regulation of medical devices is indisputable. Internationally, the growing adoption of AI-enabled technologies is likely to be among the most consequential trends in health over the next decade.^83^ However, the EU has been too long flying blind, with an intransparent and fragmented approach to addressing critical new technology advances, providing limited support to EU MedTech through this period of change. By introducing the Digital Omnibus package as well as the proposed MDR and IVDR revision in December 2025, European legislators recognized that technical sovereignty and competitiveness required changes. The remaining question is how to translate that recognition into a governance structure that delivers increased efficiency while still protecting fundamental European values and rights. Whether the EU retains the current regulatory governance structure, adopts a mixed centralised/decentralised model, or creates the most centralised medical device regulator model possible within EU-system, it must get the regulatory system to work in a coordinated manner. This would be most straightforward to achieve through a highly centralised approach. If the EU instead adopts a partially decentralised “half-way-house”, the best available path is to be aware of the model’s weaknesses and to limit their effects by taking account of approaches we suggest as the best hope for EU MedTech and provision of much needed innovations to the EU healthcare sector.

**Search strategy and selection criteria**

References for this Health Policy were identified through searches of Google Scholar and PubMed with the search terms “EU medical device regulation reform”, “centralization EU regulatory governance”, “decentralization EU regulatory governance”, “digital health regulation EU”, “European agencies legitimation”, “central authorisation”, “EU agencies delegation”, “EU agency accountability”, “future governance”, “European political strategy”, “fiscal federalism EU”, “notified bodies medical devices EU”, “FDA medical device approval”, “clinical evaluation consultation procedure”, “clinical evaluation evidence MDR IVDR”, “appropriateness of clinical data MDR”, “complex regulatory systems”, “principal-agent theory decentralization”, “risk-return trade-off centralization”, “regulatory work-sharing”, and “delegation of powers” until January 2026, and via an internet search engine search with Google using the same search terms. Only papers published in English were reviewed. The final reference list was generated on the basis of originality and relevance to the broad scope of this Health Policy.

## Contributors

A.S. and S.G. developed the concept of the manuscript. A.S. and S.G. wrote the first draft of the manuscript. A.S., T.Me., T.Mi., and S.G. contributed to the writing, interpretation of the content, and editing of the manuscript, revising it critically for important intellectual content. A.S., T.Me., T.Mi., and S.G. had final approval of the completed version. A.S., T.Me., T.Mi., and S.G. take accountability for all aspects of the work in ensuring that questions related to the accuracy or integrity of any part of the work are appropriately investigated and resolved.

## Declaration of interests

A.S. has no conflicts of interest.

S.G. is an advisory group member of the Ernst & Young-coordinated “Study on Regulatory Governance and Innovation in the field of Medical Devices” conducted on behalf of the Directorate-General for Health and Food Safety of the European Commission. S.G. has or has had consulting relationships with Una Health GmbH, Lindus Health Ltd, Flo Ltd, ICURA ApS, Rock Health Inc., Thymia Ltd, FORUM Institut für Management GmbH, High-Tech Gründerfonds Management, the European Commission Directorate General for Research & Innovation, Prova Health Ltd, Haleon Plc., Ada Health GmbH, and the Saudi Arabia Food and Drug Authority (FDA) funded through the United Nations Development Programme (UNDP), and holds share options in Ada Health GmbH. S.G. holds the following grants: BMBF 16KISA100K, HORIZON ASSESS-DHT 101137347, and HORIZON CYMEDSEC 101094218. S.G. is a News and Views Editor for npj Digital Medicine.

T.Mi. contribution to this paper was generously supported by the Novo Nordisk Foundation (NNF) via a grant for the scientifically independent Collaborative Research Program in Bioscience Innovation Law (Inter-CeBIL Program – Grant No. NNF23SA0087056). T.Mi. research was further supported by the European Union (Grant Agreement no. 101057321; the ‘CLASSICA project’). The views presented here are solely those of the authors and do not reflect those of the funders, who were not involved in the study design, in the collection, analysis, or interpretation of data, in the writing of the report; nor in the decision to submit the paper for publication. Neither the NNF nor the European Union nor the granting authority can be held responsible for them. T.Mi. consulted for Microsoft Corp. on a scientifically independent academic study of the history of risk assessments for medical devices. T.Mi. was paid by the Asian Productivity Organization (APO) for a technology transfer course in 2025; by the European Institute of Public Administration (EIPA) and the Council of the European Union for a virtual course on AI and medical device governance in 2024/25; and by the European Commission for a foresight report on emerging biotechnologies in 2024/25. T.Mi. is a steering committee member of LifeScienceLaw.dk.

T.Me. is a member of the European Medicines Agency Expert Panels for Medical Devices and Chair of the Regulatory Affairs Committee of the Biomedical Alliance.
